# Peptide receptor radionuclide therapy (PRRT) in radioiodine-refractory thyroid cancer: A case report of significant response to lu177 DOTA-TATE treatment

**DOI:** 10.20945/2359-3997000000451

**Published:** 2022-03-23

**Authors:** Saeideh Ataei-Nakhaei, Kamran Aryana, Sayyed Mostafa Mostafavi, Hadis Mohammadzadeh Kosari, Mohammad Esmatinia, Atena Aghaee

**Affiliations:** 1 Mashhad University of Medical Sciences Nuclear Medicine Research Center Mashhad Iran Nuclear Medicine Research Center, Mashhad University of Medical Sciences, Mashhad, Iran; 2 Mashhad University of Medical Sciences Faculty of Medicine Department of Medical Informatics Mashhad Iran Department of Medical Informatics, Faculty of Medicine, Mashhad University of Medical Sciences, Mashhad, Iran

## Abstract

A 59-year-old woman with follicular thyroid carcinoma underwent total thyroidectomy followed by radioiodine treatment. Following treatment, the whole-body scan did not show any abnormal radioiodine uptake. However, during the follow-up, the serum thyroglobulin (Tg) value increased without detectable thyroglobulin-antibodies. We performed a Ga-68 DOTA-TATE PET/CT showing a sternal lesion and several lung nodules with high somatostatin receptor density. Also, on the next day, FDG PET/CT was performed, which confirmed the findings. Considering the high levels of somatostatin receptor expression in such metastases, we planned lu177 DOTA-TATE therapy. After two cycles of lu177 DOTA-TATE injection, serum thyroglobulin significantly dropped, and she claimed that her sternal pain and dyspnea were much better. This was the case of a patient suffering from iodine-refractory follicular thyroid carcinoma, with somatostatin-receptor expression, treated with 177Lu-DOTA-TATE, showing a significant response.

## INTRODUCTION

The term differentiated thyroid carcinoma (DTC) encompasses papillary, follicular, and hurtle cell carcinomas of the thyroid ( 1 ). Medullary thyroid carcinoma is not included in this term. DTCs generally show favorable outcomes when treated promptly. Surgery ± radioactive iodine ablation (RIA) is the gold standard of curative treatment ( 1 ). However, some patients may lack the ability to take up radioactive iodine or even lose this ability (previous RIA-responsive cells were destroyed, but some less differentiated cells remained and progressed) as the disease progresses ( 2 ). This diminished uptake of radioactive iodine also restricts RIA-refractory patients’ survival ( 1 ). The family of somatostatin receptors was shown to regulate thyroid cells proliferation (both normal and neoplastic tissue) ( 1 ). Additionally, multiple studies detected these receptors on thyroid tumor cells. Such features suggest potential therapeutic effects for agents like 111-In-octreotide, 90Y-DOTA-TOC, and lu177 DOTA-TATE ( 1 ). Herein, we report our experience of managing an RIA-refractory FTC with lu177 DOTA-TATE therapy, which alleviated the patient’s symptoms and decreased Tg level.

## CASE REPORT

**Figure 1:**
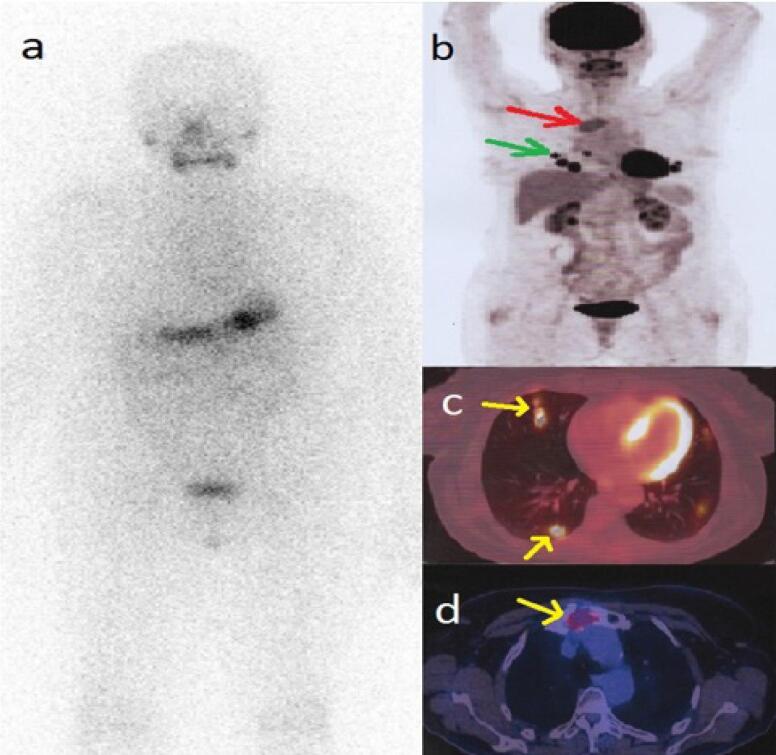
whole-body iodine scan ( **a** ) after administration of 200 mCi I-131 was negative. FDG PET/CT maximum intensity projection (MIP) ( **b** ) shows sternal and pulmonary metastases, which are more precisely localized on transaxial slices ( **c** , **d** ).

A 59-year-old woman was referred to our tertiary clinic with increased thyroglobulin (Tg) levels detected during follow-up of follicular thyroid carcinoma (FTC). She was diagnosed with FTC nine years ago (pT2N0) and treated with total thyroidectomy plus 30 millicuries (mCi) of I-131 at that point. Post-ablation whole-body iodine scan did not reveal any pathologic finding and just post surgical thyroid remnant was evident. His first serum Tg level was 0.01 ng/mL in the TSH stimulated state (serum TSH = 33); the anti-Tg antibody was absent. The neck ultrasonography was unremarkable back then. The patient had poorly adhered to her follow-up visits during that time. an empirical dose (200 mCi) of I-131 was administered, and the post-treatment scan was negative ( a ) ( Figure 1A ). On the follow-up(After 14 months), Tg level was more than 500 ng/mL with suppressed TSH. Neck ultrasonography and diagnostic I-131 scan were negative. According to the guidelines of that time and because we had just a diagnostic scan showing no iodine avidity. Rising (After 6 months) serum Tg levels (>30,000 ng/mL) were documented in the absence of anti-Tg antibody, indicating a metastatic disease. Also, the patient reported severe sternal region pain. Thus, FDG PET/CT was performed, which showed pulmonary nodules and a lytic lesion of sternal manubrium with FDG uptake ( Figures 1A-D ).

**Figure 2:**
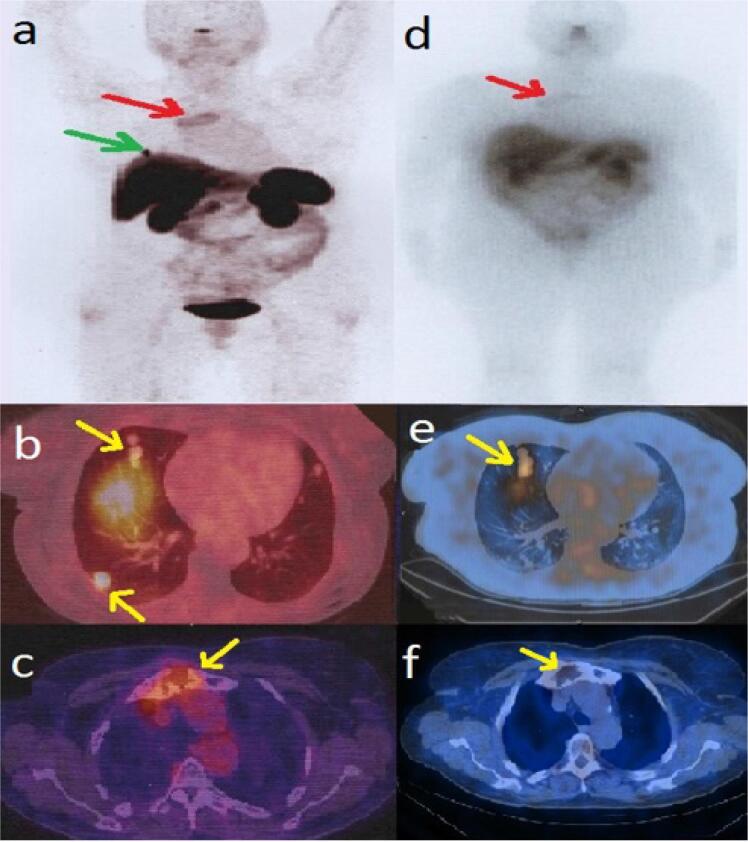
Ga-68-DOTATE MIP ( **a** ) and PET/CT ( **b, c** ) confirmed the lesions detected by FDG PET/CT. Therefore, the patient received 200 mci of lu177 DOTA-TATE and performed post-treatment WBS ( **d** ). lu177 DOTA-TATE SPECT/CT showed increased uptake in pulmonary nodules ( **e** ) and uptake in the margin of lytic lesion of the sternum **(f** ).

By performing Ga-68 DOTA-TATE PET/CT to evaluate somatostatin receptor avidity, we tried to give our patient a chance to be treated with somatostatin-receptor radiopharmaceuticals. It confirmed the findings of FDG PET/CT ( Figures 2A-C ). Therefore, the patient received 200 mci of lu177 DOTA-TATE, and a post-treatment whole-body scan was performed. The SPECT/CT images showed increased uptake in pulmonary nodules and tracer uptake in the margin of lytic lesion of the sternum ( Figures 2D-F ). Three months after the first cycle of lu177 DOTA-TATE therapy, blood analysis showed declined Tg level to 1,760 ng/mL and subsequently to 982 ng/mL two months after completing the second cycle. Additionally, the patient declared significant improvement of clinical symptoms.

## DISCUSSION

We reported a case of RIA-refractory FTC that was successfully managed via lu177 DOTA-TATE. About 25%-50% of patients with locally advanced or metastatic differentiated non-medullary thyroid carcinoma (DTC) become non-responsive to RAI ( 2 , 3 ). Treatment options are limited, one of which is recently proposed as PRRT, and there are few cases in the literature performing this treatment option ( 1 , 4 - 6 ). It has been reported that this treatment produces stabilization and even partial disease remission in these patients ( 7 ). Çinkir and Elboğa conducted a clinical study on ten thyroid cancer patients to assess the efficacy of lu177 DOTA-TATE treatment after detecting the presence of somatostatin receptors via Ga-68 labeled DOTATATE PET scan. Although she included MTC patients in her study, the overall results were promising ( 8 ). They had only one FTC stage-IV patient with no metastasis or lymph node involvement who remained stable during the follow-up. Versari and cols. performed a similar study on 11 patients, but they used 90Y-DOTATOC for PRRT; they treated three patients of FTC, two of whom remained stable, and one of them experienced disease progression ( 9 ).

In contrast to these promising outcomes, Budiawan and cols. reported 16 RIA-refractory thyroid carcinoma cases, four of which were FTC. They were treated with PRRT using 90-Yttrium, or 177-Lutetium, which showed poor long-term outcomes ( 1 ). These findings are explained mainly by these studies’ variable inclusion criteria. Çinkir and Elboğa did not include metastatic patients; however, metastasis was an inclusion feature for the Budiawan study. Although lu177 DOTA-TATE significantly reduced Tg levels and improved our patient’s sternal region pain and dyspnea, further long-term studies are needed before PRRT can be established as an option in treating RAI-refractory metastatic FTC ( 8 ).

In conclusion, lu177 DOTA-TATE therapy could be an effective alternative treatment modality in patients with elevated thyroglobulin and negative iodine scan. A sufficient somatostatin receptor expression must be confirmed in diagnostic imaging modalities like Ga-68 DOTA-TATE PET/CT scan. According to our knowledge, our case is the first RIA-resistant metastatic FTC with such a dramatic response to lu177 DOTA-TATE therapy.
